# Reference Range for the Automated Fragmented Red Cell Parameter and Its Diagnostic Utility in Red Blood Cell (RBC) Fragment Quantification: A Prospective Study

**DOI:** 10.7759/cureus.91066

**Published:** 2025-08-26

**Authors:** Ginni Bharti, Tushar Sehgal, Hemchandra Pandey, Hem C Sati

**Affiliations:** 1 Laboratory Medicine, All India Institute of Medical Sciences, New Delhi, New Delhi, IND; 2 Transfusion Medicine, All India Institute of Medical Sciences, New Delhi, New Delhi, IND; 3 Biostatistics, All India Institute of Medical Sciences, New Delhi, New Delhi, IND

**Keywords:** blood film microscopy, fragmented red blood cells, hematology analyser, hemolytic uremic syndrome (hus), laboratory automation, reference range, ttp (thrombotic thrombocytopenic purpura)

## Abstract

Background

The automated measurement of fragmented red blood cells (FRCs) is a recently introduced parameter available on modern hematology analyzers, including the XN-Series™ Automated Hematology Analyzers (Sysmex Corporation, Kobe, Japan). Its clinical relevance lies in its potential to correlate with schistocyte counts, which is a critical diagnostic step in thrombotic microangiopathies and related disorders, as their presence reflects microangiopathic red cell fragmentation and guides urgent clinical decision-making. Schistocyte quantifications are traditionally assessed manually on stained peripheral blood smears using light microscopy. However, the accuracy of FRC measurement is highly dependent on the specific technology and methodology employed by each analyzer, making the establishment of instrument-specific reference ranges essential. This study aimed to establish a reference range for FRC and to evaluate the correlation between manual schistocyte counts and automated FRC measurements.

Methods

We analyzed 179 normal samples from voluntary blood donors using the XN-Series™ automated hematology analyzer to establish the reference range for FRC. In addition, we evaluated the influence of other red blood cell (RBC) parameters on FRC measurements. To assess the diagnostic performance of FRC, we further analyzed 100 patient samples by comparing the automated FRC values with manual schistocyte counts performed on stained peripheral blood smears, using Bland-Altman analysis for agreement assessment.

Results

Based on the analysis of 179 healthy samples, the reference interval for the absolute FRC count (FRC#) was 0.0 to 0.125/µL, and for the FRC percentage (FRC%), it was 0.0 to 0.00515%. Elevated Hypo-He (hypo hemoglobin equivalent) values (red cells with hemoglobin content <17 pg) were associated with spurious increases in FRC measurements, suggesting potential analytical interference. In a separate cohort of 100 patient samples, an FRC% threshold of <1% demonstrated a negative predictive value (NPV) of 70% for excluding the presence of schistocytes on peripheral blood smear. Bland-Altman analysis comparing manual schistocyte counts with automated FRC% revealed a mean bias of 0.287, with 95% limits of agreement ranging from -2.2 to 2.8, indicating moderate agreement between the two methods.

Conclusion

Establishing reference ranges for FRC not only enhances clinical interpretation but also provides valuable feedback to hematology analyzer manufacturers for refining automated smear-generation rules. As an initial screening tool, the FRC parameter could help safely exclude over two-thirds of samples (NPV = 70%) from manual review, reducing the burden of time-intensive schistocyte quantification. Such selective flagging has the potential to improve laboratory efficiency without compromising patient safety, enabling laboratory physicians to prioritize and triage cases more effectively. The upper reference limit was well below the ≥1% schistocyte threshold for thrombotic microangiopathic anemia (TMA), supporting the role of elevated FRC as a trigger for manual smear review, which remains indispensable whenever TMA is clinically suspected.

## Introduction

Modern automated hematology analyzers provide rapid and reliable blood cell counts, supporting timely clinical decision-making [1]. Beyond standard parameters, many analyzers now offer research-use-only or extended diagnostic parameters that hold potential for broader clinical utility [1]. One such parameter is the automated fragmented red blood cell (FRC) count, available on XN-Series™ Automated Hematology Analyzers (Sysmex Corporation, Kobe, Japan), which estimates the proportion of FRCs - analogous to schistocytes observed on a peripheral blood smear [2]. 

The quantification of red blood cell (RBC) fragmentation is crucial in the diagnosis and monitoring of several microangiopathic conditions, including hemolytic uremic syndrome, thrombotic thrombocytopenic purpura (TTP), and transplantation-associated thrombotic microangiopathy (TA-TMA), where timely recognition can be lifesaving and directly influences patient management [3]. According to established guidelines, the presence of ≥1% schistocytes on a peripheral blood film (PBF) is considered a robust morphological indicator of thrombotic microangiopathic anemia (TMA) in adults [3]. However, this quantification currently depends on manual microscopic evaluation, a time-consuming and observer-dependent process.

Automated FRC measurement has emerged as a potential screening tool to complement or partially replace manual schistocyte assessment on blood smears [3-6]. While the parameter shows promise, its clinical utility, diagnostic performance, and appropriate reference ranges remain insufficiently characterized [3].

In this study, we aimed to (i) evaluate the clinical value of the automated FRC parameter using the XN-Series hematology analyzer, by comparing it to the reference manual schistocyte count on stained PBF; and (ii) establish a reference range for FRC in healthy subjects.

## Materials and methods

Patient settings

This study was a single-center, hospital-based, preliminary prospective study done on 100 leftover blood samples received in the emergency hematology laboratory of the Department of Laboratory Medicine at the All India Institute of Medical Sciences, New Delhi, India, from January 2025 to April 2025. This study was intended to provide preliminary and exploratory data on the potential clinical utility of the automated approach. Ethical approval was obtained from the Institutional Ethics Committee (approval no. IEC-1142).

Patient samples from adults aged >18 years were received from both inpatient and outpatient hospital areas and were selected based on the following criteria: red cell distribution width-coefficient of variation (RDW-CV) >14.6%, abnormal flagging for fragmented cells, or blood film requests for schistocyte/fragmented cell counting. All specimens were collected by venous puncture under proper aseptic precautions into ethylene diamine tetraacetic acid (EDTA) anticoagulated tubes (Becton Dickinson, Mountain View, CA, USA). All samples were analyzed within six hours of receipt using the XN-Series™ Automated Hematology Analyzer (Sysmex Corporation) for complete blood count and the FRC parameter. Three levels of internal quality control were performed daily on the analyzer, in accordance with laboratory protocol.

Reference interval for FRC

To establish a reference interval for FRC, we prospectively enrolled blood donors aged over 18 years who presented to the hospital for voluntary blood donation. A total of 179 donors were included, comprising 155 males and 24 females. All donors had complete blood counts within the normal reference range for hemoglobin, white blood cell (WBC) count, RBC indices, and platelet count (PLT). Their blood samples were analyzed using the XN-Series™ automated hematology analyzer (Sysmex Corporation) for the determination of both CBC and the FRC parameter. Three levels of internal quality control were performed daily on the analyzer, in accordance with laboratory protocol.

FRC measurement methodology

**Figure 1:**
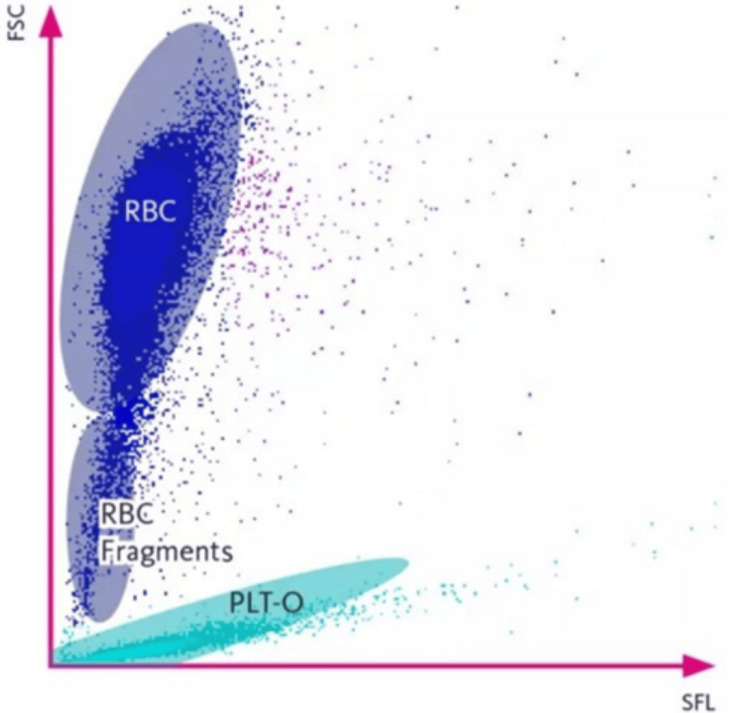
Reticulocyte (RET) scattergram The figure shows the reticulocyte (RET) scattergram, with side fluorescence (SFL) on the X-axis and forward scatter (FSC) on the Y-axis, which reflect the characteristics of both cell size and cellular content. The circled area below the red blood cell (RBC) cluster indicates the detection area for fragmented red blood cells (FRC). PLT-O reflects the optical platelet count.

FRC was measured using an automated direct method on the automated hematology analyzer, operated in reticulocyte (RET) mode. The FRC parameter is generated only when the analyzer is run in this specific mode [7]. The system employs fluorescence flow cytometry in the RET channel to quantify the percentage of FRCs (FRC%) and their absolute count (FRC#), reported as research parameters. FRCs are identified within a defined region below the RBC area on the RET scattergram (Figure 1).

Due to the absence of nucleic acids in RBCs, the intensity of the measured side fluorescence (SFL) signals is extremely low. Additionally, their high-angle forward scatter (FSC) is lower than that of intact RBCs [7].

Quantitation of schistocytes/fragmented RBCs by optical microscopy

Microscopic quantitation of schistocytes or FRCs was performed on PBFs stained with Giemsa and Leishman stains, in accordance with the guidelines recommended by the International Council for Standardization in Hematology (ICSH) [3]. The microscopy was blinded to automated results to avoid bias. The percentage of schistocytes was estimated by counting at least 1,000 RBCs in the appropriate zone of the smear - where RBCs are evenly distributed without overlapping and the feathered edge is avoided - using an optical microscope at medium magnification. Only cells with characteristic shapes, such as triangular, crescent, helmet cells, and keratocytes, were considered valid schistocytes [3].

Statistical analysis

All statistical analyses were performed using Stata version 18.0 (StataCorp LLC, College Station, TX, USA). Descriptive statistics, including mean, standard deviation (SD), median, interquartile range (IQR), and range (minimum-maximum), were calculated for both FRC absolute count and percentage. Due to the skewed distribution of values and a high frequency of zero counts, a non-parametric percentile method was employed to estimate the reference intervals. The central 95% reference range was defined by the 2.5th and 97.5th percentiles of the observed distribution. Confidence intervals for these percentiles were derived using binomial interpolation. This approach aligns with the Clinical and Laboratory Standards Institute (CLSI) EP28-A3c guidelines for establishing reference intervals in clinical laboratories and is appropriate for data that deviate from Gaussian distribution assumptions [8].

The Wilcoxon signed-rank test was used to compare the average values obtained from manual microscopy and automated FRC measurement. Bland-Altman analysis was conducted to assess the bias and 95% limits of agreement between the two methods. The intraclass correlation coefficient (ICC) was calculated to evaluate the reliability of the two approaches. A p-value of <0.05 was considered statistically significant. Simple linear regression and Pearson correlation analyses were used to assess the relationship between FRC values and other RBC parameters, including RDW-SD (red cell distribution width-standard deviation), Hypo-He (hypo hemoglobin equivalent), IRF (immature RET fraction), HFR (high fluorescence RETs), Micro R% (microcytic red cells), PLT, MCH (mean corpuscular hemoglobin), and RET percentage.

## Results

Reference range for FRC

The reference range for automated FRCs was established using the non-parametric percentile method, which defines the lower and upper limits as the 2.5th and 97.5th percentiles, respectively. For the absolute FRC#, the calculated reference interval ranged from 0.0 to 0.125/µL, while for the FRC%, the corresponding interval was 0.0 to 0.00515%.

Both FRC# and FRC% exhibited substantial right skewness, with a large proportion of individuals showing negligible or zero values. This is reflected in the measures of central tendency, where the median and IQRs for both parameters were 0.0 (IQR: 0.0-0.0). Such a distribution indicates that FRCs were either undetectable or present only in trace amounts in most healthy individuals included in the study. The mean ± SD further supports this observation, with FRC# showing a mean of 0.0071 ± 0.0256/µL and FRC% showing a mean of 0.00032 ± 0.00116%. These low mean values, coupled with relatively higher SDs, highlight the presence of occasional outliers or elevated values in a small subset of the population. This distribution pattern validates the use of percentile-based cutoffs rather than parametric estimates for reference interval determination. The confidence intervals for the 97.5th percentiles were computed using binomial interpolation, yielding a range of 0.07 to 0.151/µL for FRC# and 0.00318 to 0.00737% for FRC%. These intervals enhance the precision and reliability of the established reference ranges.

Comparison between automated FRC and microscopic schistocyte count

Of the 100 patients included in the study, 33 were male and 67 were female, with a mean age of 48.9 ± 12.44 years (range: 27-76 years). The mean schistocyte count by microscopy was 1.58%, while the mean count by the automated method (FRC) was 1.26%, with SDs of 2.89 and 2.41, respectively. The IQR was wider for microscopy (p25 = 0.3, p75 = 1.8) than for automated FRC (p25 = 0.4, p75 = 1.3) (Table 1).

**Table 1:** Summary of descriptive statistics for schistocyte counts obtained from manual microscopy and automated FRC using the Sysmex XN-9000 analyzer # indicates the Wilcoxon signed-rank test FRC, fragmented red blood cell

Method	Mean ± SD	Min-Max	Median	p25	p75	p-value#
Manual Microscopy	1.58 ± 2.89	0-26	0.87 (0-26)	0.3	1.8	0.14
Automated Count, FRC (Sysmex)	1.26 ± 2.41	0-23	0.8 (0-23)	0.4	1.3	

The Wilcoxon signed-rank test was used to compare the average values obtained by manual microscopy and automated FRC counts. The p-value was not statistically significant, indicating that the two methods are comparable.

**Figure 2:**
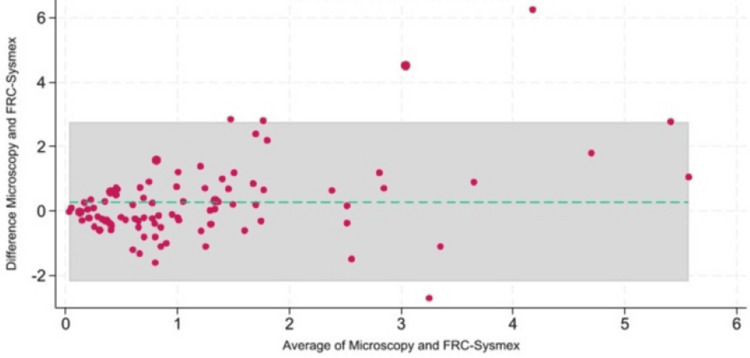
The Bland-Altman analysis to evaluate the agreement between the two methods FRC, fragmented red blood cell

Figure 2 shows the Bland-Altman analysis used to assess agreement between the methods.

It revealed a mean bias of 0.287, with 95% limits of agreement ranging from -2.2 to 2.8. While most measurements fell within these limits, 7% of the data points lay outside, indicating occasional variability. The ICC was 0.69, indicating moderate to substantial agreement between the two methods. This suggests that both microscopy and automated FRC provide reasonably consistent results. The sensitivity of automated FRC compared to the microscopic gold standard was 69% (95% CI: 52-83%), while specificity was 46% (95% CI: 33-59%). The area under the curve (AUC) was 0.58 (95% CI: 0.47-0.67), with a negative predictive value (NPV) of 70%. Table 2 shows the correlation between FRC and other red cell parameters

**Table 2:** Correlation between FRC and other red cell parameters RDW-SD, red cell distribution width-standard deviation; RDW-CV, red cell distribution width-coefficient of variation; Hypo-He, hypo hemoglobin equivalent; IRF, immature reticulocyte fraction; HFR, high fluorescence reticulocyte; Micro R, micro red cells; PLT, platelet count; MCH, mean corpuscular hemoglobin; RET, reticulocyte percentage

Predictor Variable	Regression Equation (Y = a + bx)	Correlation Coefficient (R)	p-value	R-squared (R²)
RDW-SD	y = -0.4121 + 0.0113 × RDW-SD	0.117	<0.001	0.0137
RDW-CV	y = -2.1914 + 0.1612 × RDW-CV	0.4236	<0.001	0.1795
Hypo-He	y = 0.0700 + 0.0527 × Hypo-He	0.5002	<0.001	0.2502
IRF	y = -0.1784 + 0.0271 × IRF	0.2419	<0.001	0.0585
HFR	y = -0.0091 + 0.0653 × HFR	0.2725	<0.001	0.0743
Micro R	y = 0.0408 + 0.0346 × Micro R	0.3673	<0.001	0.1349
PLT	y = 0.0008 + 0.0006 × PLT	0.0768	0.01	0.0059
MCH	y = 4.7571 - 0.1432 × MCH	-0.3587	<0.001	0.1287
RET	y = -0.0400 + 2.1963 × RET	0.1028	<0.001	0.0106

## Discussion

Automated FRC counts may reflect schistocyte counts obtained from stained PBFs examined under a microscope [6]. FRCs can now be quantified using newer generations of complete blood count analyzers, particularly those from Siemens (ADVIA series) and Sysmex (XE- and XN-series). However, the technology used to measure FRCs varies between manufacturers [9]. Several studies have demonstrated a good correlation between the automated FRC parameter and schistocyte percentages in patients with TMA or in post-transplantation settings [6].

In our study, we compared the automated FRC method on the Sysmex XN-series analyzer with manual microscopic schistocyte counts and found that the manual method yielded higher values. A similar observation was reported by Banno et al. using the Sysmex XE-series [6]. This discrepancy may be attributed to interobserver variability inherent in manual microscopy. Conversely, Lesesve et al. reported that the ADVIA 120 analyzer (Bayer HealthCare, Tarrytown, NY, USA) tended to overestimate schistocyte counts compared to microscopy [10]. In our analysis, the NPV of the automated FRC parameter was 70% for excluding the presence of schistocytes. Such a performance suggests that FRC could serve as a reliable “rule-out” parameter, enabling laboratories to safely bypass manual PBF examination for a substantial proportion of samples. Given the labor-intensive and operator-dependent nature of schistocyte quantification, incorporating FRC-based screening thresholds into analyzer flagging algorithms could streamline workflow, reduce turnaround time, and optimize resource allocation, while maintaining diagnostic safety. However, the moderate NPV also underscores that FRC alone should not replace manual smear review in high-risk clinical scenarios, such as suspected TMA, or when clinical suspicion remains high despite low FRC values. This finding is consistent with previous reports in the literature [3], reinforcing the potential utility of FRC as an initial screening tool.

In this study, we established the reference range for FRC, and a comparative Table 3, summarizing these findings alongside data from the existing literature, is presented [11].

**Table 3:** Comparison of reference ranges for fragmented red cells (FRCs) reported in published studies

Reference	Center	Analyzer	Number	Population	Median/Mean ± SD	Min-Max (95% CI)
Jiang et al. (2001) [2]	Kobe, Japan	XE-2100	762	Adults	ND	0.03-0.56
Lesesve et al. (2012) [9]	Roma, Italy	XE-2100	146	Adults	0.33/0.69 ± 0.74	0.2-2.8
Lesesve et al. (2012) [9]	Nancy, France	XE-2100	232	Adults	0.34/0.36 ± 0.13	0.05-0.65 (0.31-0.37)
Lesesve et al. (2012) [9]	Thionville, France	XE-5000	111	Adults	1.1/0.33 ± 0.15	0-2.9
Abe et al. (2009) [12]	Mie, Japan	XE-2100	120	Adults	0.04	0-0.2
Sehgal et al. (2025) (this study)	Delhi, India	XN-9000	179	Adults	0.00/0.007 ± 0.03	0-0.16 (0.003-0.010)

We also observed a moderate correlation between increased Hypo-He and FRCs, aligning with results reported by Lesesve et al. [11]. Hypo-He refers to the percentage of RBCs with a hemoglobin content lower than 17 pg. It serves as a metric for hypohemoglobinized RBCs and is defined by an MCH <17 pg (reference interval for adult MCH: 26-32 pg) [13]. These findings underscore the importance of cautious interpretation of FRC values, particularly in patients with abnormal Hypo-He values or instrument-generated flags. Currently, Sysmex recommends implementing automated rules for blood film preparation based on specific flags. For FRC, a threshold of >1% is used to trigger slide preparation. We suggest that incorporating Hypo-He into the decision rule may improve the specificity of smear review triggers [10].

As FRCs may include very small microcytic red cells, we also evaluated the relationship between Micro R% and FRC. Lesesve et al. reported no artefactual impact of increased Micro R% on FRC values in control samples [10]. Similarly, our data suggest that Micro R% does not significantly influence FRC%. In Table 4, we outline a proposed clinical pathway for systematically managing false-positive results from automated FRC measurements. These pathways integrate stepwise triage based on analyzer flags, correlation with clinical and laboratory data, targeted manual smear review when indicated, rule-out strategies for low-risk cases, feedback-driven refinement of analyzer thresholds, and training initiatives to ensure accurate interpretation and appropriate clinical use of FRC.

**Table 4:** Proposed clinical pathway for managing false-positive FRC flags FRC, fragmented red blood cells; CBC, complete blood count; RBC, red blood cell; LDH, lactate dehydrogenase; PBF, peripheral blood film; TMA, thrombotic microangiopathy

Step	Pathway Component	Key Actions
1	Initial Analyzer Flag (If FRC exceeds the predefined cut-off)	Flag the sample for further review; Correlate with patient history, diagnosis, and suspicion of TMA or other causes (e.g., mechanical heart valves, burns, infections).
2	Secondary Automated Parameters Review	Evaluate CBC indices (RBC morphology flags, platelet count, LDH, bilirubin, reticulocyte count); Review hemolysis index from chemistry analyzers, if available.
3	Rapid Morphological Confirmation	Perform manual PBF examination only in flagged cases with supportive clinical/lab indicators present; Use a two-tier microscopy approach-(quick low-power scan for fragments, followed by detailed quantification if fragments suspected) in high workload.
4	Rule-Out Algorithm for Low-Risk False Positives	If no clinical suspicion of TMA and normal labs, retest the same sample or request a fresh draw before manual smear review.
5	Feedback Loop to Analyzer Rules	Document confirmed false positives; Use findings to periodically review and adjust analyzer flagging thresholds with the manufacturer.
6	Education and Awareness	Train laboratory staff and clinicians on contextual interpretation of FRC; Avoid over-reliance without morphological confirmation.

Strengths

This study is among the few to establish instrument-specific reference ranges for both absolute FRC (FRC#) and percentage FRC (FRC%), using a well-characterized healthy donor population. Analysis was performed using the Sysmex XN-9000, an advanced and widely adopted hematology analyzer, thereby enhancing the reproducibility and clinical applicability of the findings. The use of Bland-Altman analysis enabled a systematic comparison between automated FRC values and manual schistocyte counts, offering valuable insights into their level of agreement and diagnostic utility. The study also identified the influence of RBC indices - particularly elevated Hypo-He values - on FRC measurements, a key observation with practical implications for laboratory interpretation. Inclusion of both healthy donors and a patient cohort facilitated the evaluation of both reference intervals and real-world diagnostic performance, increasing the translational relevance of the study. Finally, the application of standardized methodologies, including peripheral smear examination under light microscopy and robust statistical analysis, adds rigor and credibility to the results.

Limitations

This study had certain limitations. The reference ranges established are specific to the Sysmex XN-series analyzer and may not be generalizable to other hematology platforms employing different methodologies or algorithms for FRC detection. The healthy donor samples used for reference intervals may not fully represent the broader healthy population. The diagnostic performance analysis was based on a small (n = 100), high-risk cohort, which may not be sufficiently powered to capture the full spectrum of conditions associated with schistocytosis - such as thrombotic microangiopathies or mechanical hemolysis - and may limit the generalizability of metrics such as NPV to unselected populations.

## Conclusions

We established reference ranges for FRC in healthy individuals. While FRC remains a research-use parameter and is not yet routinely reported outside the laboratory, it shows promise as an initial screening tool - potentially excluding over two-thirds of samples from manual smear review. This selective flagging could reduce the burden of schistocyte quantification, improve laboratory efficiency, and support effective case triage. Importantly, the upper reference limit observed in our healthy cohort was well below the ≥1% schistocyte threshold recommended for the diagnosis of TMA, reinforcing the potential utility of elevated FRC values as a trigger for manual PBF examination. Nonetheless, manual smear review remains essential in all cases with clinical suspicion of TMA, regardless of FRC values.
